# The maximum size of cell-aggregates is determined by the competition between the strain energy and the binding energy of cells

**DOI:** 10.1016/j.heliyon.2024.e40560

**Published:** 2024-11-20

**Authors:** Francesco Gentile

**Affiliations:** Nanotechnology Research Center, Department of Experimental and Clinical Medicine, University Magna Graecia of Catanzaro, 88100, Catanzaro, Italy

**Keywords:** Cell aggregates, Cell-cell interactions, Biological systems, Maximum cluster size, Tissue engineering

## Abstract

The development of tissues and organs is affected by how cells interact with each other to form aggregates. Such an interaction is in turn determined by several different factors, such as inter-cellular attractive forces, cell motility, and the strain energy of cells. Here, we have used mathematical modelling and numerical simulations to explore how the interplay between these factors can influence the formation and stability of 2D cell aggregates. Cell aggregates were created by incrementally accumulating cells over an initial seed. The binding energy density of these aggregates was determined using the harmonic approximation and was integrated into a probabilistic model to estimate the maximum cluster size, beyond which the aggregate becomes unstable and breaks into smaller fragments. Our simulations reveal that the ratio of strain energy to internal adhesive energy ($U_{s}/U_{b}$) critically impacts cell aggregation; smaller ratios allow for larger cluster sizes. These findings have significant implications for tissue engineering, in-vitro modeling, the study of neurodegenerative diseases, and tissue regeneration, providing insights into how physical and biological characteristics of cells influence their aggregation and stability.

## 1 Introduction

The formation, growth and development of tissues and organs depends on a large extent on the interaction forces between cells. The magnitude of such forces and the competition between cell-cell interactions and cell motility can be a major driver of cell aggregation dynamics [[1], [2], [3], [4], [5], [6], [7]]. When plated on a substrate, cells cluster into groups with a finite size, rather than forming continuous, homogenous, indefinitely large aggregates. A number of reports have highlighted that on both adhesive and non-adhesive surfaces, the size and area of cell colonies and aggregates reach a steady state value [[8], [9], [10]], where such an asymptotic value depends on the cell line, culture conditions, the characteristics of the plating surface. Some of these reports have explored the evolution dynamics of 2D cultures of neurons on, *remarkably*, nanoscale surfaces [11,12]. In experiments where neuronal cells have been cultured on random rough surfaces [11] and zinc oxide nanowires [12], researchers have observed the emergence of superclusters of cells with a maximum average size of about 200 cells per cluster.

Since the performance of biological systems is influenced by how their constituents interact [[13], [14], [15], [16], [17]], and by the number of these interactions, an estimate of the maximum cluster size in 2D and 3D cell aggregates may be important in tissue engineering and in-vitro-model applications, the study and analysis of neurodegenerative diseases, tissue regeneration and repair.

**Fig. 1:**
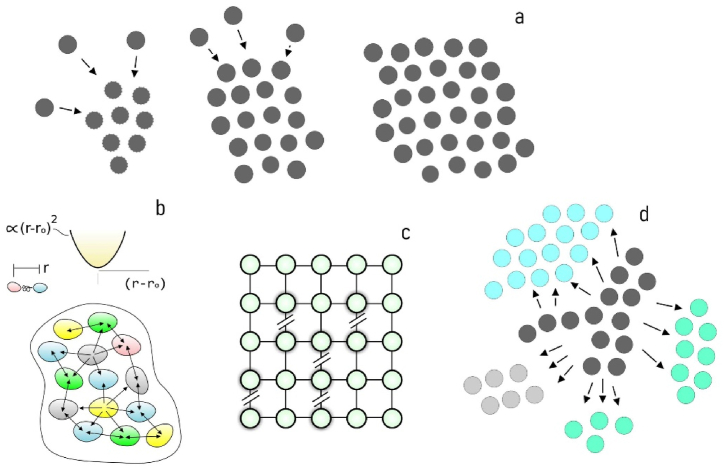
*Model schematics*. To determine the internal energy of aggregates, we created clusters of cells with increasing cluster size (***a***). We then used an harmonic-force potential model to represent the binding energy between interacting cells (***b***). Such an energy was then integrated into a probabilistic model to estimate the maximum cluster size (***c***), beyond which the aggregate becomes unstable and breaks into smaller fragments (***d***).

In this work, we have used mathematical modelling and numerical simulations to investigate the role of cell-cell interactions and strain energy on the maximum size of 2D aggregates of cells. To do this, by incrementally accumulating individual cells over an initial seed, we created aggregates of cells with a tunable cluster size (Fig. 1a). Then, using the harmonic approximation (Fig. 1b) we determined the *binding energy density* of the systems as a function of their size, and used this value in a probabilistic model that estimates the maximum allowed cluster size, i.e. the size of the cluster for which the strain energy of cells overtakes the adhesive potential energy of the system (Fig. 1c). For this critical value, the system experiences failure and the originating aggregate breaks into smaller fragments (Fig. 1d). In simulations where the asymptotic internal adhesive energy of the cluster ($U_{b}$) and the strain energy of cells ($U_{s}$) were varied over large intervals, we found that cell aggregation critically depends on the ratio $U_{s}/U_{b}$, and the smaller $U_{s}/U_{b}$, the greater the maximum allowed cluster size.

This research was inspired by the observation that the size of atomic nuclei is determined by the competition between forces: the nuclear force, a short-range attractive force, and the electromagnetic force, a long-range repulsive force. In the same way, here we explored whether, *for a fixed value of cell-density*, the stability of a cluster of cells could depend on two sole parameters: the cell-cell attractive binding energy and the cell strain energy, i.e. the biological characteristics of cells.

## 2 Methods

We generated clusters of closely interacting cells with a variable cluster size. Then, for each considered cluster size and configuration, we calculated the binding energy density of the cluster and compared such a value to the strain energy of cells. *The strain energy is the maximum value of energy that cells can develop and use to escape the potential well that ties them together*. When values of the strain and of the binding energy are unbalanced, the cluster becomes unstable, and it is likely to split into smaller elements. The maximum cluster size is the dimension of an aggregate of cells for which more than half of the cells exhibit values of strain energy higher than binding energy.

### 2.1 Cell-cell binding energy

The potential energy of adhesion between cells is given by the *superposition* of the chemical potentials of *individual* cell-cell bridges:

(1) $$\mu_{i}=\mu_{o}+\frac{1}{2}\gamma_{i}l_{i}^{2}\text{,}$$

where $\mu_{o}$ is a constant, $l_{i}$ is the length of the cell bridge stretching, and $\gamma_{i}$ is the spring constant for stretching of the bridges. Thus, the internal energy of cell-cell bridges depends quite critically on the cell-cell separation distance. Considering that the typical value of the spring constant for a cell-bridge ranges from $10^{-2}$ to 10mN/m, and that the total number of receptors involved in the binding of a cell with another is approximately $10^{2}$, then the sum of the ${\gamma_{i}}^{\prime }s$ over the number of receptors $\left(\gamma =\sum_{bonds}\gamma_{i}\right)$, i.e. the harmonic force constant, varies from 1mN/m to $10^{3}\;mN/m$ [18,19]. Notice that these values of harmonic force constant are consistent with recent experimental reports [20,21]. In the same way, one can find the ground energy value (e) as $e=\sum_{bonds}\mu_{o}$. The ground energy value is the energy of adhesion of a cell with another when the separation distance is set to zero, it oscillates between $10^{-3}\;pJ$ and 1pJ [22,23].

Thus, interaction between cells is here approximated by a harmonic-potential energy function of the type

(2) $$u_{ij}=e+\frac{1}{2}\gamma \;l^{2},l<\delta_{co}\text{,}$$

where subscripts i and j indicate definite cells within the cluster, and e and γ have been described above. In Eq. (2): $l=d_{ij}-r_{o}$ denotes the Euclidean distance between cells $\left(d_{ij}\right)$ displaced by equilibrium length $\left(r_{o}\right)$. The equilibrium length (offset) $r_{o}$ is the value of distance where the energy between cells achieves a minimum. Moreover $\delta_{co}$ is a cutoff distance: the maximum allowed interaction length between pairs of cells. The total potential energy of the system is then determined as the sum of all individual energy elements $\Psi =\sum_{i\neq j}u_{ij}$.

The harmonic approximation used in this study is accurate in the short cell-cell distance range, and less accurate otherwise. It serves as the best mathematical approximation of the effective potential of intercellular interaction, of which the Lennard-Jones potential represents the most accurate physical representation [[24], [25], [26], [27], [28], [29], [30]]. Notably, the harmonic approximation underestimates repulsive forces when the intercellular distance is significantly smaller than the cell diameter and overestimates attractive forces when the distance is much larger than the cell diameter. However, in the configurations considered for this study: (i) Short-range interactions are excluded due to the method of generating numerical cell aggregates (see methods section 2.3). (ii) Long rage interactions are excluded because of the cut-off distance $\delta_{co}$ introduced in the model specifically for this purpose.

Thus, the harmonic approximation used in this model is an acceptable assumption. It remains the simplest model for exploring biological systems with many components, providing an adequate description of the system, still ensuring computational tractability.

In the rest of this research, the cutoff distance $\delta_{co}$ was set to 10 cell diameters, equivalent to 100μm. The value of $\delta_{co}$ was chosen in consideration of true physical dimensions of cellular protrusions, of filipodia, and other intercellular bridges, as reported in the specialized literature. In particular, filopodia are thin, actin-rich structures protruding from the lamellipodial actin network [[31], [32], [33]]. They are involved in a variety of cellular processes, including cell migration and adhesion. Notably, filopodia extend up to 35μm, and occasionally more than 70μm [31]. Other cellular links, including tunnelling nanotubes and intercellular bridges, can reach a length of 200μm [34]. Further to this end, a number of studies [35,36] have illustrated that cells can communicate mechanically by forces transported through the extracellular matrix over distances up to 200μm. Moreover, in the case of neuronal cells [37], axons stretch out for several hundreds of microns. However, while in this study we have used the specific value of 10 cell diameters, the cutoff distance is a model parameter that can be conveniently adjusted to accommodate different cell types and junctions. By varying the value of $\delta_{co}$, one can influence the energy content of the system and thus the form of the specific adhesion energy ψ, discussed later in the paper. Diagrams reported in a Supplementary Material 1 illustrate the effects of $\delta_{co}$ on the characteristics of the cell aggregate. They describe how, depending on $\delta_{co}$, and on the remaining model parameters *e* and γ, one can generate configurations of cells that are indefinitely large, that have a finite size, or that are composed by loosely spaced cells, without structure.

### 2.2 Kinetic energy of a cell

The kinetic energy of a cell can be readily calculated using the equation $k_{i}=m\;v^{2}/2$, where m is the mass of the cell and v is its velocity. The mass of a cell can vary from approximately $10^{-11}\;g$ for a red blood cell [38] to $10^{-9}\;g$ for a mammalian cell [39]. The velocity of a displacement of a cell on a substrate varies significantly depending on cell type, cell density, and substrate characteristics. In examining the motility of eukaryotic cells on a gelatin gel, Alamo and collaborators found that the mean velocity of a cell can be as high as 15μm/min [40]. For HaCaT cells growing in a mono-layer configuration on collagen, Selmeczi and colleagues measured a migration velocity ranging from 10 to 40 μm/h [41]. Even considering, for the estimate of k, the largest possible values of cell-weight of $10^{-9}\;g$ and cell velocity of 15μm/min, the kinetic energy of migration of that cell would be $k_{i}∼10^{-25}\;J$. This vanishingly small figure is some $10^{12}$ times smaller than the chemical, specific adhesion energy between cells, and is thus neglected in the following of this research.

The aim for this section, was showing that the contribution of kinetic energy to the overall energy of a system of cells is vanishingly small, and thus, that it can be disregarded. To illustrate this, we used the upper range of cell mass and velocity values commonly reported in the literature. So that, even if data are not homogeneous, they give an overestimation and possibly an upper limit of the kinetic energy of a cell. Thus, if the kinetic energy for this hypothetical cell is insignificant, it would be even more so for real cells with lower mass and velocity values. However, we recognize the need of a more specific and more rigorous approach. For this, we provide here a more precise estimate of the kinetic energy of some cells of interest for those working the biomedical engineering field. We will consider epithelial cells first. The velocity of epithelial cells has been reported to vary from 0 to a maximum of 50μm/h, with an average value of about 12μm/h [[42], [43], [44]]. At the same time, the mass of epithelial cells [45] varies in the $10^{-10}÷10^{-9}\;g$ range. Thus, the kinetic energy, for these, ranges from $k∼{5\times 10}^{-31}\;J$ to ${k∼5\times 10}^{-28}\;J$. In the case of fibroblasts [46], for which $m=2.\;40\times 10^{-10}÷3.70\times 10^{-9}\;g$ and v=8÷12μm/h, one has $k∼{5\times 10}^{-31}÷{2\times 10}^{-29}\;J$, similar to epithelial cells. Even considering neuronal cells, with a mass that can be as large as $m=2\times 10^{-6}\;g$ [45] and a velocity v∼150μm/h [47,48] one would have an estimation of kinetic energy $k∼10^{-24}\;J$, still much smaller than the energy associated to specific cell-cell interactions. Thus, data validate the initial assumption of not considering the kinetic energy of cells.

### 2.3 Generating numerical cell-aggregates

We generated a random distribution of N points on a surface. Points were represented in a Euclidean space with an orthonormal basis set using the standard notation x=(x,y). Coordinates of the points were sampled from a uniform random distribution from a sampling space of length $N^{1/2}\;\left(2\;r\right)\;\alpha$, where 2r is the cell diameter and $\alpha =1.8\;\sqrt{2}$ is a model parameter. Points of the aggregates were generated iteratively. Each new $i^{th}$ randomly generated point was accepted if its minimal Euclidean distance from the existing dataset $\left(l_{\min}\right)$ was comprised within the 2.2−4 cell radius interval: $2.2\;r<l_{\min}<4\;r$. *In doing so, we avoid cell overlapping, still guaranteeing that cell aggregates are sufficiently compact*. The generalization of cell distribution by a random process is just a mathematical technique to generate cell aggregates. The constants in the model, such as the lattice constant and the coefficient of variation, allow obtaining cell distributions where cell nuclei are not precisely placed on a surface following periodic or regular artificial patterns, but are instead randomly scattered similar to real cell cultures.

### 2.4 Cell strain energy

The strain energy $\left(U_{s}\right)$ is the mechanical work that cells exert on a surface during adhesion and migration [49]. It is tightly interwoven to the biological processes of cells such as protrusion and contraction of the cell's body, and retraction of the rear [40,50], that are associated, in turn, to the dynamics of the actin cytoskeleton and of the substrate adhesion sites. Values of $U_{s}$ are generally smaller than the metabolic energy of cells, and vary in the 0.1−10 fJ range. $U_{s}$ is a quantitative estimate of the cell work output - it is the net positive energy that cells can develop over time, in contrast to the negative binding energy of adhesion. The ratio between the cell strain energy and cell-cell adhesion energy controls the stability of a cluster of cells, and is a key determinant in cluster division.

## 3 Results

### 3.1 Total and specific binding energy of a cluster of cells

**Fig. 2:**
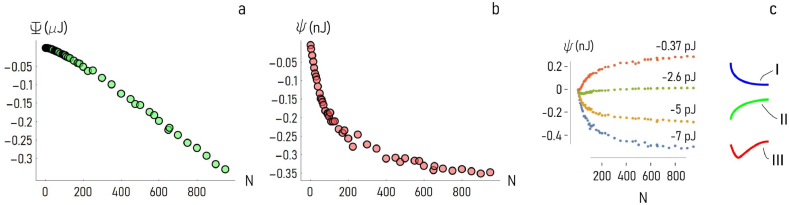
*Total and specific binding energy of a cluster*. The harmonic force model described in the methods of the article was used to estimate the total (***a***) and specific (***b***) energy of a cluster of cells, as a function of cluster size. The specific binding energy (ψ) shows a very high sensitivity to the ground energy of the harmonic-potential model. Depending on the parameters of the model, the shape of the ψ(N) curve can take different forms, and classified as type I, II or III (***c***), reflecting the different behaviors of cells in the aggregate.

Using the methods described in a separate section, we generated closely interacting arrangements of cells in a plane, where the number N of cells in the cluster was varied in the 2−950 interval. We then determined the total *binding* energy of the cluster (Ψ) for each considered cluster size and values of the harmonic potential model parameters, i.e. the ground value of the potential e and the harmonic force constant γ. The total energy was determined as the sum of the individual binding energies among cell pairs in the cluster. Fig. 2a illustrates how Ψ varies as a function of N, for fixed values of e=−3.6pJ and γ=0.31mN/m. Notably, Ψ steadily decreases with N, indicating that the *overall* adhesive energy of a cluster increases with the size. Values of Ψ vary from Ψ∼0μJ for N=2, to Ψ∼−0.33μJ for N=950. If one divides the values of total binding energy by the size of a cluster, he obtains the specific energy of adhesion of the system, i.e. the *binding energy* (ψ=Ψ/N). The binding energy is the smallest amount of energy required to remove a cell from the cluster. Differently from Ψ, in the low dimensional range ψ steeply decreases with N. Then, for sufficiently high cluster sizes, it attains to a steady state value that, for the present configuration, is ψ∼−0.35nJ (Fig. 2b). Thus, simulations suggest that there exists a *critical cluster size*, above which the incremental gain of specific energy is zero. However, the form of the ψ(N) curve may depend on the constants of the model. Fig. 2c reports ψ as a function of N, for different values of the ground energy value varying from e=−7pJ to e=−0.36pJ, and for a constant γ∼1mN/m. Depending on e, the specific energy of adhesion function takes different forms. For small values of e, ψ steadily decreases with N until it reaches a steady-state, negative value of energy. For high values of e, ψ steadily increases with N until it reaches a steady-state, positive value of energy. For intermediate values of e, ψ decreases quickly in the beginning, reaches a lowest value, then it increases with N. In the following of the work, we will designate systems-of-cells exhibiting similar behaviors: of type I, II and III, respectively (Fig. 2c). Thus, without any external intervention or power source, type I aggregates are likely to growth indefinitely, differently from type II systems, whose formation is generally inhibited. Type III cell-aggregates will assume a configuration with a finite and definite cluster size, i.e. the value of N for which ψ is minimized.

### 3.2 Effect of the ground energy (e) and of the harmonic force constant (γ)

**Fig. 3:**
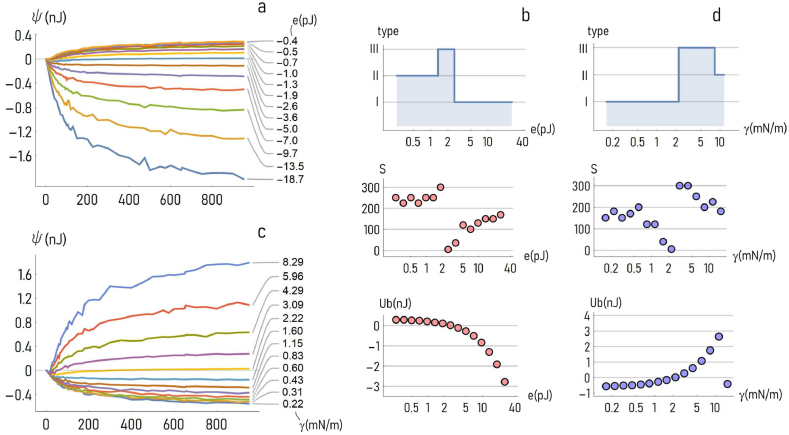
*Effect of the model parameters*. The specific binding energy ψ(N) shows a very high sensitivity to the model parameters, including the ground energy e (***a***–***b***) and the harmonic force constant γ (***c***–***d***). Diagrams in the insets ***b***-***d*** illustrate how the shape of the ψ curve transitions from class I to class II, to class III, and how the critical cluster size (S) and the steady-state value of ψ ($U_{b}$) vary as a function of e (***b***) and γ (***d***).

To dissect how the parameters of the harmonic-potential model influence the shape of ψ, we performed a number of simulations where the levels of the ground energy (e) and the values of the harmonic force constant (γ) were varied over large intervals. In a first simulation campaign, for a fixed γ∼1mN/m the values of e were changed from −18pJ to −0.4pJ. Results of the simulations illustrate that, as e moves from more negative (−18pJ) to less negative (−0.4pJ) values, the shape of the ψ(N) curve changes significantly, with the values of ψ steadily transitioning from −1.8nJ to 0.4nJ (Fig. 3a). Remarkably, for some values of e (−2.2pJ) the slope of ψ switches from negative to positive – and the formation of clusters of cells turns from being spontaneous to being energetically unfavorable. Remarkably, the ψ(N) curves can be described by three parameters:

(i) *The critical cluster size* (S). The value of N for which ψ attains the 66% of its steady state value.
(ii) *The asymptotic specific binding energy of cells* ($U_{b}$). The steady state value of ψ.
(iii) *The type of the curve* (type). It delineates the tendency of cells to either assemble (type I) or not (type II) into clusters, or to form clusters with a finite size (type III), as discussed in a previous section.

Fig. 3b is a log-linear plot showing how the type, the critical cluster size and the specific binding energy of cells vary with the *absolute* value of the ground energy e, i.e. the adhesion strength. For e moving from ∼0.4 to ∼18pJ, the type transitions from type II to type I, passing through type III. For the present configuration and the values of the constants, the transition occurs for a value of e of about e∼2pJ. Notably, above this limit the critical cluster size S correlates positively with e, while the binding energy of a cluster decreases with e. Thus, the larger the cell-cell adhesion strength, the larger the size above which the binding energy of a cluster of cells reaches a steady state, and the larger the value of the steady state, i.e. the intra-cluster binding energy levels. The effects of the harmonic force constant γ on ψ are reported in Fig. 3c. Here, the values of the specific energy of adhesion ψ in a cluster of cells are reported as a function of N for a fixed ground energy level (e∼5pJ) and for values of the spring constant varying from γ∼0.2mN/m to γ∼8mN/m. For the considered values of the parameters, as the values of the spring constant increase, the shape of the ψ(N) curve gradually evolves from type I to type II, with a transition occurring at about γ∼2mN/m, as evidenced by the log-linear diagram in Fig. 3d. Below the 2mN/m spring constant limit, defining type I systems, the critical cluster size *decreases* for increasing values of γ, while the steady-state binding energy states move from lower (more stable) to higher (less stable). Thus, compliant cell-cell bridges with low values of γ (lower than 2mN/m) boost the formation of cell aggregates. In contrast, for stiff cell-cell bridges with high values of γ (higher than 2mN/m), the emergence of cell clusters is possible at some metabolic cost – i.e. the positive-valued $U_{b}$ reported in the diagram of Fig. 3d.

### 3.3 Coincidental effects of e and γ on cell clustering

**Fig. 4:**
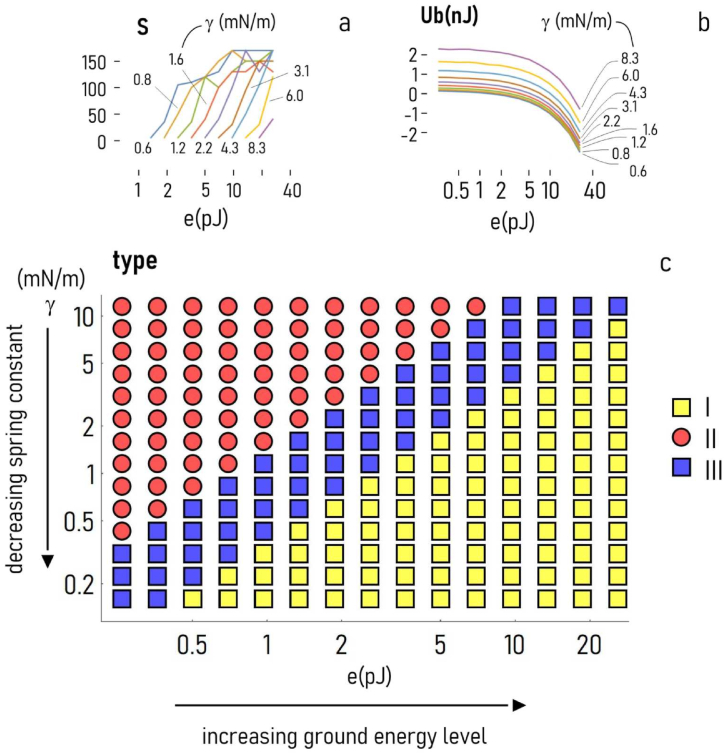
*Coincidental effects of model parameters*. The diagram illustrates how the critical cluster size (S) (***a***) and the asymptotic value of the specific binding energy ($U_{b}$) (***b***) depend on different combinations of e and γ. The diagram in inset (***c***) shows how the type of an aggregate depends on e and γ, not-separately. For larger values of e and lower values of γ, the specific binding energy of cells is described by a type I curve, an hallmark of cell clustering.

Collectively, results of the simulations indicate that both the critical cluster size and the *asymptotic* binding energy are affected by e and γ. The critical cluster size S - the minimal dimension of a group of cells upon condensation - increases with e and decreases with γ (Fig. 4a). In the same way: $U_{b}$, the steady state value of energy that binds cells to a cluster, correlates positively with e, and negatively with γ (Fig. 4b), meaning that the larger e and the lower γ, the higher the adhesion strength of a cell to an aggregate. For certain combinations of e and γ - $U_{b}$ becomes positive, meaning that the forces in the system turn from attractive to repulsive, a no-condensation condition. The log-log plot in Fig. 4c illustrates how the specific binding energy characteristics and shape of a cluster vary as a function of e and γ. Similar to the phase diagrams used in physical chemistry and engineering, the design map in Fig. 4c provides indication of how key variables of a system determine the system's state. For the combinations of the parameters used in this study, we can identify three regions in the diagram. For values of e and γ such that γ<0.44e we have type I systems, with a high probability of cell condensation (γ is here expressed in mN/m, e in pJ). For values of e and γ such that γ>1.88e we have type III systems, where cell clustering is hampered. In an intermediate region (1.88e>γ>0.44e) we have type III system, with possible cell condensation.

## 4 Discussion

### 4.1 Effect of size

Results of the simulations suggest that values of the *specific binding energy* ψ of cells in a cluster can possibly determine the evolution of the cluster. ψ, in turn, depends on the physical and biological characteristics of the cells, such as e and γ. Moreover, ψ is a function of N: ψ(N). Thus, the size of a cluster controls its internal energy. However, there is another way by which the dimension of an aggregate may influence its stability: a *probabilistic size effect*.

We hypothesize, in analogy with the scaling laws on material strength used in mechanics [51,52], that the larger the sample size the larger the likelihood that at least one of its elements (*cells*) will present an energy level higher than the cell strain energy $U_{s}$.

In an aggregate of N cells with specific binding energy ψ and strain energy $U_{s}$, the probability of *none* of the cells of generating energy with an intensity higher than ψ is $\exp\left(-N\left(U_{s}/\psi \right)\right)$. Accordingly, in the same system the probability of having at least one cell with strain energy higher than ψ is:

(3) $$p=1-e^{-N\left(\frac{U_{s}}{\psi }\right)}\text{,}$$

where ψ, the specific binding energy, is here taken in absolute value. Results shown in Fig. 3, Fig. 4 suggest that ψ varies with N with a law of the type:

(4) $$\psi \left(N\right)=U_{b}\left(1-e^{-\beta N}\right)\text{,}$$

where β is a model parameter. Combining equations (3), (4), and solving for N, one obtains the size of an aggregate for which there is a probability p of having at least one element with an energy higher than the specific binding energy:

(5) $$N_{p}\left(p,\zeta \right)=-\frac{\log\left(1-p\right)}{\zeta }+\frac{\;\omega \left({\frac{\beta }{\zeta }\;\left(1-p\right)}^{\frac{\beta }{\zeta }}\;\log\left(1-p\right)\right)}{\beta }\text{,}$$

where $\zeta =U_{s}/U_{b}$ and ω is the product-logarithm Lambert function.

The probability of having, in the aggregate, z cells with an energy higher than $U_{b}$ is $p_{z}={p_{1}}^{z}$, from which it immediately follows $p_{1}={p_{z}}^{1/z}$. Substituting back this value in Equation (5), one obtains the recursive function $N_{p}\left({p_{z}}^{1/N_{p}},\zeta \right)$: an estimate of the size of a cluster in which z cells have the energy necessary to escape the cluster with a probability $p_{z}$. With β=1 and z=N/2, this association becomes:

(6.1) $$N_{p\left(N/2\right)}=-\frac{1}{\zeta }\;\log\left(1-p^{2\;\frac{\zeta }{\;\sigma }}\right)+\omega \left({\left(1-p^{2\;\frac{\zeta }{\;\sigma }}\right)}^{\frac{1}{\zeta }}\;\log\left(1-p^{2\;\frac{\zeta }{\;\sigma }}\right)\right)\text{,}$$

where,

(6.1) $$\zeta =\frac{U_{s}}{U_{b}}$$

(6.2) μ=1−p

(6.3) $$\vartheta =\frac{\mu^{\frac{1}{\zeta }}\;\log\;\mu }{\zeta }$$

(6.4) σ=ζω(ϑ)−log(μ)

Equation (6) is an estimate of the size of a cluster for which *half* of its elements have a probability greater than or equal to p to escape from the cluster. Assuming that this is a necessary and sufficient condition for an aggregate of cells to disintegrate, Equation (6) represents therefore a good approximation of the maximum cluster size with a given probability p.

**Fig. 5:**
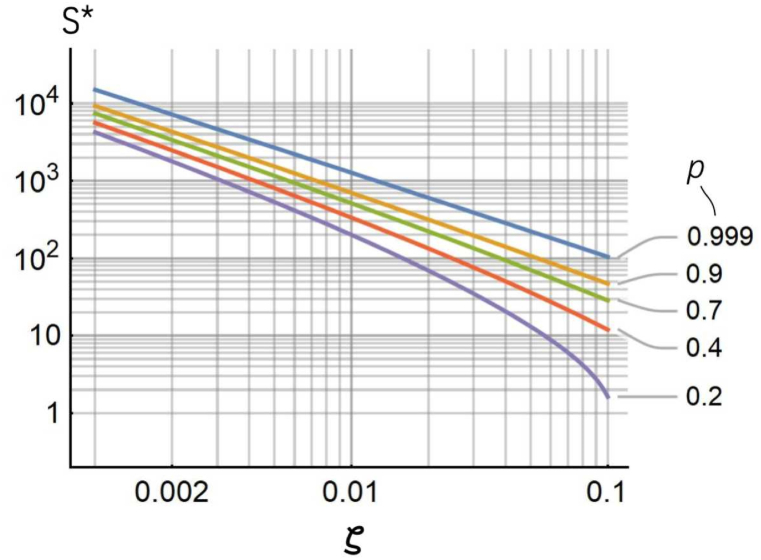
*Maximum cluster size*. The diagram illustrates how the maximum allowed cluster size $S^{*}$ varies as a function of the ratio of the strain energy to the binding energy, $\zeta =U_{s}/U_{b}$, for different values of a threshold probability p.

The maximum cluster size ($S^{*}$) depends thus on the parameter $\zeta =U_{s}/U_{b}$ and on p. The log-log diagram in Fig. 5 illustrates how $S^{*}$ varies as a function of ζ for several values of p. The smaller ζ and the higher p, the larger $S^{*}$. For a fixed p=0.9, $S^{*}$ transitions from $S^{*}∼50$ cells for ζ=0.1, to $S^{*}∼700$ cells for ζ=0.01, to $S^{*}∼4300$ cells for ζ=0.002. Thus, Equation (6) predicts that cell aggregates can disaggregate into smaller parts even for low (lower than one) $U_{s}/U_{b}$, provided that the cluster is sufficiently large. For cells with a strain energy in the 1−10 fJ range, and a specific binding energy in the 1−100 pJ range, the maximum allowed cluster size varies from 700 (ζ=0.01) to more than $10^{6}$ cells (ζ=0.0001) (p=0.9).

### 4.2 Effect of the substrate

Notably, the model that we have developed does not contemplate interaction forces with the substrate. While this study and a more sophisticated evolution of the model are left for future work, we can nevertheless make some observations:

i. Many of the conclusion of the work rely on the binding energy density ψ, a function of cluster size: ψ(N). Interaction with a surface would affect all cells in a cluster in the same way - since all cells in cluster are exposed to the substrate in the same way. This means that, following interaction with a surface, the specific energy of the system would increase/decrease of a constant quantity. This in turn means that the diagrams relevant for this research and reported in **Figures** from **2** to **3** would undergo a *rigid translation*, without changing the essence and the main findings of the work.
ii. Thus, interaction/adhesion with an external substrate would have, depending on the surface characteristics, as an effect an enhancement or a reduction of the cluster asymptotic specific binding energy ($U_{b}$). Used in the probabilistic model described by Equation (6), this updated value for $U_{b}$ would cause the size of the cluster to deviate from the value predicted without surface interaction. If $U_{b}$ increases (decreases) following adhesion with the substrate, then the maximum allowed cluster size would also increase (decrease).

Thus, the model template that we have developed and Equation (6), in fact, implicitly incorporate the effects of the surface. By tailoring the geometrical, and physical-chemical characteristics of the material on which cells are cultured, one can regulate the binding energy $U_{b}$, thus impacting on $S^{*}$. Low-adhesive surfaces with lower values of $U_{b}$ and higher ζ enhance cell condensation (Fig. 5). This can possibly explain the formation of connected cell-networks and organoids on hydrophobic, or moderately hydrophilic, PDMS (polydimethylsiloxane) surfaces [[53], [54], [55], [56]].

### 4.3 Effect of the extracellular medium

This study was designed to examine under what conditions a system of cells forms clusters, especially focusing on cell-colonies. In the context of cell culture, a colony is a cluster of identical cells, clones, on a surface with or without a medium. The medium, in turn, represents the extracellular environment where cells move, contributing to regulating the structures and functions of cells. Such a medium, in particular, provides an array of physical stimuli to the cell, such as stiffness, fluid shear stress and hydraulic pressure [57]. These stimuli are in part determined by the extracellular fluid viscosity. The effects of viscosity can be readily incorporated into the model as an additive, constant energetic term, υ. This term adds up to the cell strain energy $U_{s}$, such that $U_{s}\to U_{s}+\upsilon$. The reason why the extracellular matrix viscosity can be modeled as a constant, is that viscosity influences cell motility independently on cell position. Differently, for instance, from cell binding interactions that relevantly depend on cell distance. Thus, the extracellular matrix effects can be taken in account without changing the structure of the model: instead, it is sufficient tuning the model parameters. This approach is similar to the methods previously applied in the case of the effects of the substrate.

The increased viscosity of the medium has, as a result, a reduced motility of cells. In fact, the extracellular viscous fluid behaves as a mechanical damper that converts kinetic energy into thermal energy. Thus, for effect of viscosity, the cell strain energy decreases: the previously introduced viscous term, υ, is negative. A reduced value of strain energy and thus of ζ $\left(\zeta =U_{s}/U_{b}\right)$ yields, through Equation (6), to an increase of cluster size, for fixed values of $U_{b}$ and p, as evidenced in the Supplementary Fig. 2.

Notably, the prediction of the model that an increase in viscosity is associated with an increase in cluster size is consistent with independent reports and research. In Ref. [58], it is illustrated that the number of Human-Hepatoma spheroids in soft microcapsules decreases, and their size increases, for increasing values of viscosity of the microcapsules. In the same paper it is demonstrated that cells in viscous media exhibit increased levels of F-actin, facilitating cell spreading and aggregation. Further to this end, the study review reported in Ref. [59] indicates that increased matrix viscosity can enhance cell aggregation and clustering. Specifically, it promotes the formation of spheroids due to the dissipation of cell-generated traction forces, contrasting with elastic matrices, which lead to cell spreading. This suggests that higher viscosity encourages more clustered cell formations. Moreover, research results reported in Ref. [60] indicate that viscous media promote cooperation among bacterial cells.

### 4.4 Identification of the model parameters

Many of the results of the work rely on two sole model parameters: $U_{b}$, the asymptotic value of specific cell-cell energy, and $U_{s}$, the strain energy of cells. Both of these parameters can be tuned by experiments, as outlined below and described in a separate Supplementary Material 3.

One can determine $U_{b}$ if he knows e, γ and $\delta_{co}$ of a cell. Characteristics values of intercellular adhesion energy can be found either in literature (see for example reference [30] for mouse blastocyst cells, reference [61] for L. lactis cells, reference [62] for U87MG glioma cells), or determined experimentally. The experimental measurement of cell-cell adhesion energy can be accomplished by several methods: among which, notably, atomic force microscopy (AFM) and optical tweezers (OT).

Similarly to cell adhesion energy, values of $U_{s}$ can be determined indirectly from the measurement of the traction forces exerted by a cell on a surface [40,[63], [64], [65]], from the measurement of the deformation of the surface, and then multiplying the two. In some cases, values of the strain energy of cells are available in literature. For example, for eukaryotic cells [40].

Thus, while this study was theoretical in nature and was not focused on the experimental determination of the energy of cellular systems, however the model allows for the precise estimation of parameter values from empirical data. Therefore, it is not merely a conceptual exercise but a practical tool for accurately predicting the behavior and evolution of a system of cells of the same type on 2D surfaces.

## 5 Conclusions

We have calculated the specific, internal adhesive energy ψ in clusters of closing interacting cells as a function of cluster size. We observed that ψ shows a very high sensitivity to the ground energy level e and the spring constant γ of the harmonic potential model describing the interaction between individual cells. For *low* local energy minimum levels and *low* values of stiffness, ψ is negative, meaning that cell condensation is permitted. Conversely, for values of e approaching zero and high values of γ, ψ is positive, a no-cell-condensation condition. However, clusters with negative, attractive energies are not necessarily boundless. Using a probabilistic model developed in analogy to the scaling-laws on material strength, we found that there exists a size $S^{*}$, above which cluster growth is energetically unfavorable. This maximum allowed cluster size depends on the strain energy $U_{s}$ and the binding energy $U_{b}$, the biological characteristics of a cell, in a combined fashion: $\zeta =U_{s}/U_{b}$. Such that the lower ζ, the larger the steady state size of an aggregate of cells. Findings of this study have implications in tissue engineering, tissue regeneration and repair, the development and test of organoids and in-vitro-models, the study of neurodegenerative disorders.

**Table:** List of symbols

variable	significance
N	Number of cells in a cluster
r	radius of a cell.
$\delta_{co}$	Cut off value. It indicates the maximum interaction length between pairs of cells.
e	Ground value of the potential.
$r_{o}$	Equilibrium length.
γ	Harmonic force constant.
Ψ	Total internal energy of an aggregate of cells.
ψ	Specific internal energy of an aggregate of cells.
$U_{b}=\lim_{N\to \infty }\psi \left(N\right)$	Steady state value of ψ.
$U_{s}$	Cell strain energy.
$\zeta =U_{s}/U_{b}$	Ratio between the strain energy and the steady-state binding energy.
S	Critical cluster size.
$S^{*}$	Maximum cluster size.
$N_{p}\left(p,\zeta \right)$	Size of an aggregate for which there is a probability p of having at least one element with an energy higher than $U_{b}$.

## Data availability statement

All data and related metadata are available upon reasonable request.

## Declaration of competing interest

The author declares no conflict of interest.
