# Supplementary material for: The maximum size of cell-aggregates is determined by the competition between the strain energy and the binding energy of cells
Source: Heliyon. 2024 Nov 20;10(23):e40560. doi: 10.1016/j.heliyon.2024.e40560 (PMC11625300; doi:10.1016/j.heliyon.2024.e40560)
Supplement: Multimedia component 1 [file mmc1.docx]

**The maximum size of cell-aggregates is determined by the competition between the strain energy and the binding energy of cells**

Francesco Gentile ^1^, *

^1^ Nanotechnology Research Center, Department of Experimental and Clinical Medicine
University of Magna Graecia, 88100 Catanzaro, Italy

* francesco.gentile@unicz.it

**Supplementary Material**

**Supplementary Material 1**. *Effect of the cutoff distance*.

In the research study, the maximum cell-cell interaction distance, i.e. the cutoff distance $\delta_{co}$, was fixed as 10 cell diameters, $\delta_{co}=10 d$, i.e. $100 \mu m$. The value of $\delta_{co}$ was chosen in consideration of true physical dimensions of cellular protrusions, of filipodia, and other intercellular bridges, as reported in the specialized literature. In particular, filopodia are thin, actin-rich structures protruding from the lamellipodial actin network [1-3]. They are involved in a variety of cellular processes, including cell migration and adhesion. Notably, filopodia extend up to $35 \mu m$, and occasionally more than $70 \mu m$ [2]. Other cellular links, including tunnelling nanotubes and intercellular bridges, can reach a length of $200 \mu m$ [4]. Further to this end, a number of studies [5, 6] have illustrated that cells can communicate mechanically by forces transported through the extracellular matrix over distances up to $200 \mu m$. Moreover, in the case of neuronal cells [7], axons stretch out for several hundreds of microns.

However, while in this study we have used the specific value of 10 cell diameters, the cutoff distance is a model parameter that can be conveniently adjusted to accommodate different cell types and junctions. By varying the value of $\delta_{co}$, one can influence the energy content of the system and thus the form of the specific adhesion energy $\psi$. Diagrams in the **Supplementary Figure 1** illustrate the effects of $\delta_{co}$ on the characteristics (type) of the cell aggregate. Specifically, the diagrams show how, depending on $\delta_{co}$, the remaining model parameters $e$ (ground energy value) and $\gamma$ (spring constant) generate a configuration of cells that is indefinitely large (type I), that has a finite size (type III), or that is composed by loosely spaced cells, without structure (type II).

Results illustrate that, in the same e-$\gamma$ space, the larger the value of cut-off, the larger the number of type II configurations. Thus, increasing the intercellular distance decreases the probability of finding large clusters, possibly because cells are exposed to an increased number of intercellular forces that tend to break the systems into smaller elements.


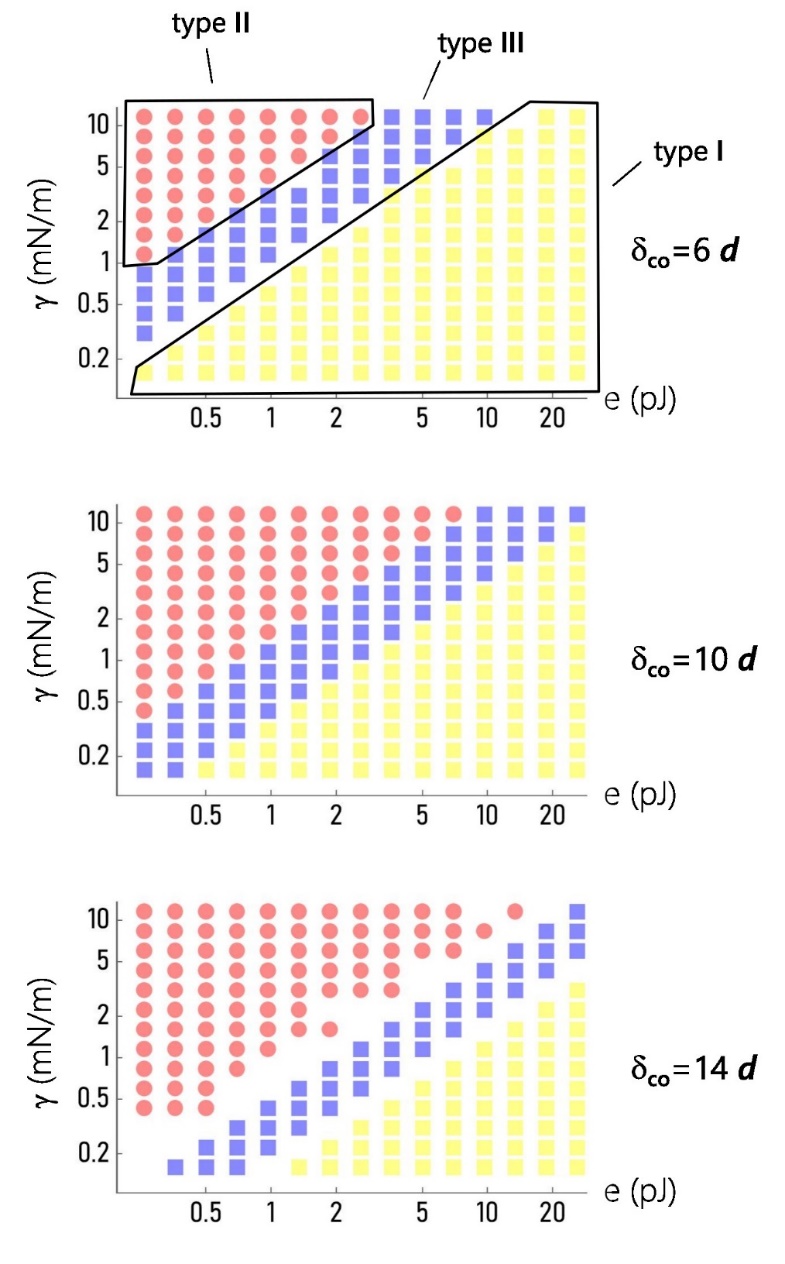


**Supplementary Figure 1.** The effects of $\delta_{co}$ on the characteristics of the cell aggregate.

**Supplementary Material 2**. *Effect of the extracellular medium*.

This study was designed to examine under which conditions a system of cells forms clusters, especially focusing on cell-colonies. In the context of cell culture, a colony is a cluster of identical cells, clones, on a surface with or without a medium. The medium, in turn, represents the extracellular environment where cells move, contributing to regulating the structures and functions of cells. The **Supplementary Figure 2**, reported below, describes the effects of viscosity of the medium on cell motility.


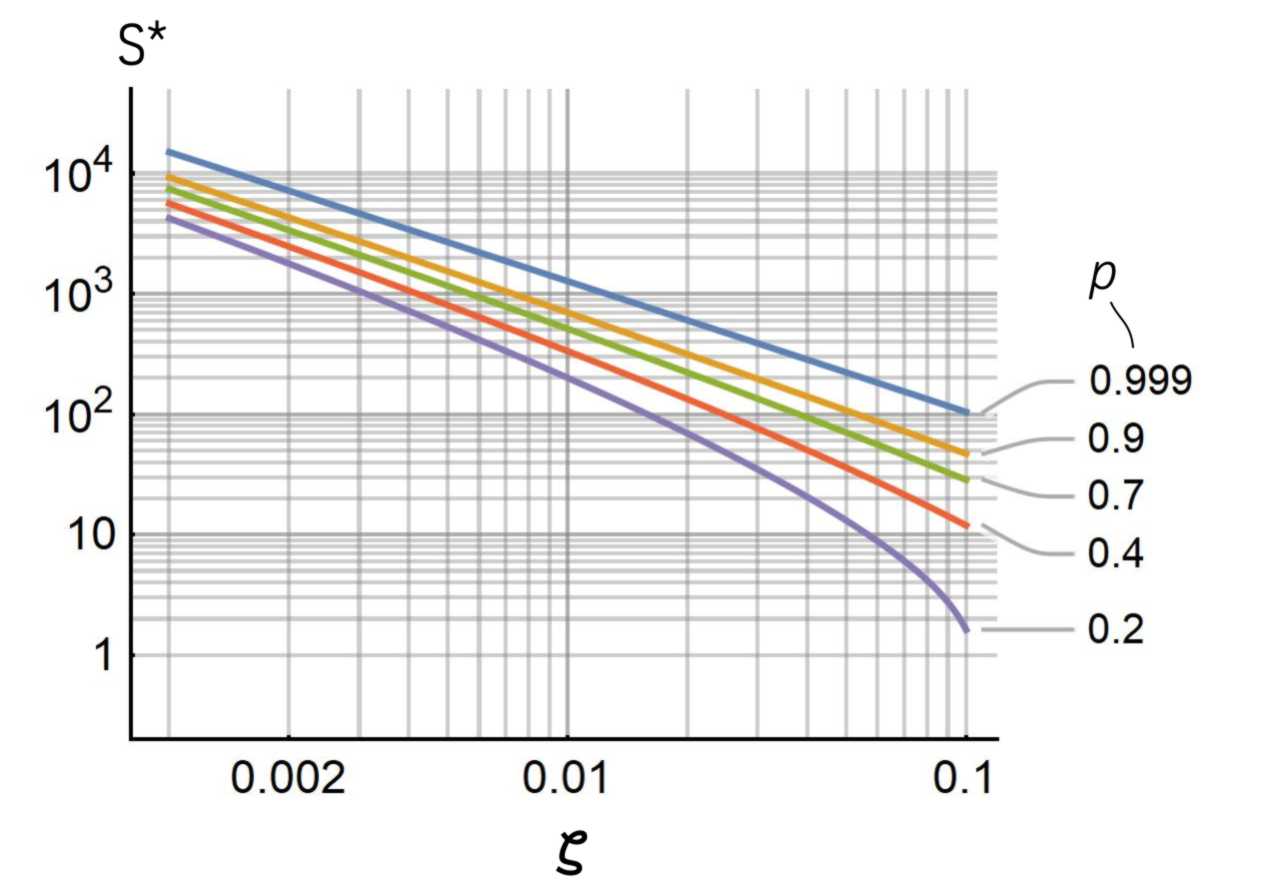


$S\uparrow$

$\zeta\downarrow$

**Supplementary Figure 2**. The extracellular viscous fluid behaves as a mechanical damper that converts kinetic energy into thermal energy. Consequently to an increase of viscosity, the cell strain energy $U_{s}$ decreases, and so does the model parameter $\zeta$. This, in turn, through equations 6 of the main text, leads to an increase of cluster size, for fixed values of $U_{b}$ and $p$.

**Supplementary Material 3**. *Identification of the parameters*.

Many of the results of the work rely on two sole model parameters: $U_{b}$, the asymptotic value of specific cell-cell energy, and $U_{s}$, the strain energy of cells. We will focus on these parameters separately, describing how they can be retrieved from literature or determined by experiments

*3.1 The asymptotic value of specific cell-cell energy* $U_{b}$*.*

The specific cell-cell adhesion energy is determined as the sum of the potential energy between cell-pairs, divided by the number of cells in a cluster:

| $\psi=\frac{1}{N}\sum_{pairs} u_{ij}$ | (S.1) |
| --- | --- |

where

| $u_{ij}=e+\frac{1}{2}\gamma l^{2}.$ | (S.2) |
| --- | --- |

Thus, if one elaborates on Equations S.1 and S.2, for sufficiently large values of $N$ (i.e. the asymptotic limit of $\psi$), he obtains:

| $U_{b}=e+\frac{1}{2}\gamma\left\langle l^{2} \right\rangle$ | (S.3) |
| --- | --- |

where $e$ is the ground value of the harmonic potential that describes cell-cell interactions, $\gamma$ is the harmonic force constant, and $\left\langle l^{2} \right\rangle$ is the mean squared cell-cell length, averaged all over the cell-cell distances smaller than the cut off limit (maximum interaction length) $\delta_{co}$. Equation (S.3) can be readily approximated as:

| $U_{b}\sim e+0.2 \gamma\delta_{co}^{2}$. | (S.4) |
| --- | --- |

Thus, one can determine $U_{b}$ if he knows $e$, $\gamma$ and $\delta_{co}$ of a cell. Characteristics values of intercellular adhesion energy can be found either in literature (see for example reference [8] for mouse blastocyst cells, reference [9] for L. lactis cells, reference [10] for U87MG glioma cells), or determined experimentally.

The experimental measurement of cell-cell adhesion energy can be accomplished by several methods: among which, notably, atomic force microscopy (AFM) and optical tweezers (OT).

*3.2 Measuring cell-cell adhesion energy by AFM and OT.*

Using AFM [11-15], adhesion energy is typically measured by functionalizing the AFM tip with a single cell, either by chemically or biologically attaching the cell to the cantilever tip. In some cases, the AFM tip can be functionalized with molecules, such as ligands or antibodies, that mimic cell-cell adhesion proteins. The AFM cantilever is then brought into contact with a target cell, which is immobilized on a substrate or held in place by another method. As the cantilever approaches, the cell on the tip forms bonds with the target cell. After the interaction is established, the cantilever is retracted, and the forces exerted as the cantilever pulls away from the cell are measured. This interaction produces a force-distance curve that reveals key information about where adhesion begins, where it breaks, and the magnitude of the forces involved. When the AFM tip retracts, the peak in the force measurement corresponds to the maximum adhesion force, which reflects the strength of the bonds. If multiple bonds are present, the force curve may show several rupture events before the cells completely separate. The adhesion energy itself, representing the work required to separate the cells, is calculated from the area under the force-distance curve.

| $u=\int\boldsymbol{F}\cdot\boldsymbol{s} d\boldsymbol{s}$ | (S.5) |
| --- | --- |

There are several important considerations when using AFM to measure adhesion energy. First, the AFM cantilever must be calibrated to convert the raw deflection data into actual force units, typically in piconewtons. Accurate calibration is essential for obtaining meaningful results. Additionally, the amount of time the AFM tip remains in contact with the cell can influence adhesion strength, as longer contact durations allow more adhesion molecules to engage. The type of adhesion molecules involved is also crucial, with proteins such as integrins or cadherins often playing a central role in cell-cell adhesion.

OTs [16-18] offer a different approach for measuring adhesion energy. This technique uses highly focused laser beams to trap and manipulate individual cells or beads that are coated with cell adhesion molecules. By precisely controlling the movement of the trapped cell or bead, it can be brought into contact with a second cell, where adhesion bonds can form. After the cells are allowed to interact, the optical trap is used to pull the cells apart, with the force exerted by the trap being recorded as the cells are separated. Similar to AFM, the force-displacement data from this process produces a force-distance curve, which can be analyzed to calculate the energy required for detachment.

The forces measured by optical tweezers are typically calibrated based on the laser’s intensity and the physical properties of the trapped object. The maximum force recorded during detachment is considered the peak adhesion force. Adhesion energy is determined from the area under the force-distance curve, much like in AFM. If the contact area between the cells is known, the adhesion energy can be normalized per unit area, providing further insight into the strength of cell-cell interactions.

When comparing AFM and optical tweezers for measuring adhesion energy, each technique has its strengths. AFM is particularly valuable for detailed analysis of adhesion mechanics, offering high-resolution force measurements and the ability to simultaneously capture topographical data on the cells. It is ideal for measuring stronger adhesion forces, but the direct contact between the AFM cantilever and the cells can potentially alter the cells' properties. In contrast, optical tweezers provide a non-invasive approach, making them excellent for studying live cells in a more natural state. While optical tweezers are best for measuring weaker adhesion forces, they offer less detailed mechanical information than AFM.

*3.3 Other methods for determining cell adhesion energy.*

Notably, in addition to AFM and OT, there are other reported methods [19] to estimate cell-adhesion energy. Traction Force Microscopy (TFM) [12, 8], is used for studying the forces cells exert on their surroundings, such as the extracellular matrix (ECM) or neighboring cells. It relies on embedding fiducial markers into a deformable substrate, which are displaced as cells exert forces on the substrate. These displacements are then used to infer the traction forces exerted by cells. TFM is often combined with confocal microscopy to enable high-resolution, three-dimensional measurements of force distribution across cells. It has been extensively used to study cellular contractility and adhesion in a variety of biological systems. Micropipette Aspiration [20] is one of the earliest techniques used to measure cell-cell adhesion forces. In this method, cells are partially aspirated into a micropipette, and the force required to detach them from another cell or surface is measured. The force of adhesion can be inferred from the pressure applied to the cell. Micropipette Aspiration has been used to study cadherin-mediated adhesion in embryonic tissues and other cell types. It is often employed in studies involving soft tissues or cells with well-defined adhesion properties, such as endothelial cells. Dual Micropipette Aspiration (DPA) [19] is an extension of micropipette aspiration, where two micropipettes are used to manipulate two cells simultaneously. This allows for the measurement of forces directly involved in cell-cell adhesion by bringing two cells into contact and then measuring the force required to separate them. DPA has been used to study interactions in various biological systems, including cancer metastasis, where cell-cell adhesion plays a critical role in the ability of cancer cells to detach and migrate. Magnetic Tweezers [19] are similar to optical tweezers but use magnetic fields to manipulate magnetic beads attached to cells or molecules. By applying a controlled magnetic field, researchers can exert forces on cells and measure their response. Magnetic tweezers are particularly useful for studying mechano-transduction, where mechanical signals are converted into biochemical signals in the cell. This technique has been applied in the study of integrin-mediated adhesion, providing insights into how cells respond to mechanical forces in their environment.

*3.4 The strain energy of cells,* $U_{s}$*.*

The strain energy of cells ($U_{s}$) is the second model parameter that, combined with $U_{b}$, determines through Equation (6) in the main text cell-cluster fate. The strain energy refers to the mechanical work cells apply to a surface during adhesion and migration [21]. It is closely linked to key cellular processes such as the protrusion and contraction of the cell body and the retraction of the rear [22, 23], which are driven by the dynamics of the actin cytoskeleton and adhesion sites on the substrate. $U_{s}$ provides a quantitative measure of the work cells perform over time, representing the net positive energy generated, in contrast to the negative binding energy of adhesion. The balance between strain energy and cell-cell adhesion energy is crucial for maintaining the stability of a cell cluster and plays a key role in determining whether the cluster will divide.

Similarly to cell adhesion energy, values of $U_{s}$ can be determined indirectly from the measurement of the traction forces exerted by a cell on a surface [24, 22, 25-27], from the measurement of the deformation of the surface, and then multiplying the two. In some cases, values of the strain energy of cells are available in literature. For example, for eukaryotic cells [22].

Thus, while this study was theoretical in nature and was not focused on the experimental determination of the energy of cellular systems, however the model allows for the precise estimation of parameter values from empirical data. Therefore, it is not merely a conceptual exercise but a practical tool for accurately predicting the behavior and evolution of a system of cells of the same type on 2D surfaces.

**References**

1. Mattila PK, Lappalainen P. Filopodia: Molecular architecture and cellular functions. Nat Rev Mol Cell Biol 2008 -06;9(6):446–54. DOI: 10.1038/nrm2406.

2. Jacinto A, Wolpert L. Filopodia. Current Biology 2001 -08-21;11(16):R634. DOI: 10.1016/S0960-9822(01)00378-5.

3. Medalia O, Beck M, Ecke M, Weber I, Neujahr R, Baumeister W, Gerisch G. Organization of actin networks in intact filopodia. Current Biology 2007 -01-09;17(1):79–84. DOI: 10.1016/j.cub.2006.11.022.

4. Korenkova O, Pepe A, Zurzolo C. Fine intercellular connections in development: TNTs, cytonemes, or intercellular bridges? Cell Stress 2020 01/07/;4(2):30–43. DOI: 10.15698/cst2020.02.212.

5. Nahum A, Koren Y, Ergaz B, Natan S, Miller G, Tamir Y, Goren S, Kolel A, Jagadeeshan S, Elkabets M, Lesman A, Zaritsky A. Inference of long-range cell-cell force transmission from ECM remodeling fluctuations. Commun Biol 2023 -08-03;6(1):811. DOI: 10.1038/s42003-023-05179-1.

6. Davidson CD, Midekssa FS, DePalma SJ, Kamen JL, Wang WY, Jayco DKP, Wieger ME, Baker BM. Mechanical intercellular communication via matrix-borne cell force transmission during vascular network formation. Adv Sci (Weinh) 2024 -01;11(3):e2306210. DOI: 10.1002/advs.202306210.

7. Hagemann C, Moreno Gonzalez C, Guetta L, Tyzack G, Chiappini C, Legati A, Patani R, Serio A. Axonal length determines distinct homeostatic phenotypes in human iPSC derived motor neurons on a bioengineered platform. Adv Healthc Mater 2022 -05;11(10):e2101817. DOI: 10.1002/adhm.202101817.

8. Koyama H, Okumura H, Otani T, Ito AM, Nakamura K, Kato K, Fujimori T. Effective mechanical potential of cell–cell interaction in tissues harboring cavity and in cell sheet toward morphogenesis. Front Cell Dev Biol 2024 -07-22;12. DOI: 10.3389/fcell.2024.1414601.

9. Feuillie C, Formosa-Dague C, Hays LMC, Vervaeck O, Derclaye S, Brennan MP, Foster TJ, Geoghegan JA, Dufrêne YF. Molecular interactions and inhibition of the staphylococcal biofilm-forming protein SdrC. Proc Natl Acad Sci U S A 2017 -04-04;114(14):3738–43. DOI: 10.1073/pnas.1616805114.

10. Arawi DE, Vézy C, Déturche R, Lehmann M, Kessler H, Dontenwill M, Jaffiol R. Advanced quantification for single-cell adhesion by variable-angle TIRF nanoscopy. Biophysical Reports 2021 -12-08;1(2):11–21. DOI: 10.1016/j.bpr.2021.100021.

11. Sancho A, Vandersmissen I, Craps S, Luttun A, Groll J. A new strategy to measure intercellular adhesion forces in mature cell-cell contacts. Sci Rep 2017 April 10,;7(1):46152. DOI: 10.1038/srep46152.

12. Chala N, Zhang X, Zambelli T, Zhang Z, Schneider T, Panozzo D, Poulikakos D, Ferrari A. 4D force detection of cell adhesion and contractility. Nano Lett 2023 -04-12;23(7):2467–75. DOI: 10.1021/acs.nanolett.2c03733.

13. Dufrêne YF. Atomic force microscopy, a powerful tool in microbiology. Journal of Bacteriology 2002 Oct;184(19):5205. DOI: 10.1128/JB.184.19.5205-5213.2002.

14. Li QS, Lee GYH, Ong CN, Lim CT. AFM indentation study of breast cancer cells. Biochem Biophys Res Commun 2008 -10-03;374(4):609–13. DOI: 10.1016/j.bbrc.2008.07.078.

15. Evans EA, Calderwood DA. Forces and bond dynamics in cell adhesion. Science 2007 -05-25;316(5828):1148–53. DOI: 10.1126/science.1137592.

16. Català-Castro F, Schäffer E, Krieg M. Exploring cell and tissue mechanics with optical tweezers. Journal of Cell Science 2022 -08-09;135(15):jcs259355. DOI: 10.1242/jcs.259355.

17. Favre-Bulle IA, Scott EK. Optical tweezers across scales in cell biology. Trends in Cell Biology 2022 -11-01;32(11):932–46. DOI: 10.1016/j.tcb.2022.05.001.

18. Arbore C, Perego L, Sergides M, Capitanio M. Probing force in living cells with optical tweezers: From single-molecule mechanics to cell mechanotransduction. Biophys Rev 2019 -10-01;11(5):765–82. DOI: 10.1007/s12551-019-00599-y.

19. Khalili AA, Ahmad MR. A review of cell adhesion studies for biomedical and biological applications. Int J Mol Sci 2015 -08-05;16(8):18149–84. DOI: 10.3390/ijms160818149.

20. Benoit M, Selhuber-Unkel C. Measuring cell adhesion forces: Theory and principles. Methods Mol Biol 2011;736:355–77. DOI: 10.1007/978-1-61779-105-5_21.

21. Cox BN, Smith DW. On strain and stress in living cells. Journal of the Mechanics and Physics of Solids 2014 -11-01;71:239–52. DOI: 10.1016/j.jmps.2014.07.001.

22. Del Alamo JC, Meili R, Alonso-Latorre B, Rodríguez-Rodríguez J, Aliseda A, Firtel RA, Lasheras JC. Spatio-temporal analysis of eukaryotic cell motility by improved force cytometry. Proc Natl Acad Sci U S A 2007 -08-14;104(33):13343–8. DOI: 10.1073/pnas.0705815104.

23. Bastounis E, Meili R, Álvarez-González B, Francois J, del Álamo JC, Firtel RA, Lasheras JC. Both contractile axial and lateral traction force dynamics drive amoeboid cell motility. J Cell Biol 2014 -03-17;204(6):1045–61. DOI: 10.1083/jcb.201307106.

24. Butler JP, Tolić-Nørrelykke IM, Fabry B, Fredberg JJ. Traction fields, moments, and strain energy that cells exert on their surroundings. Am J Physiol Cell Physiol 2002 -03;282(3):595. DOI: 10.1152/ajpcell.00270.2001.

25. Maskarinec SA, Franck C, Tirrell DA, Ravichandran G. Quantifying cellular traction forces in three dimensions. Proc Natl Acad Sci U S A 2009 -12-29;106(52):22108–13. DOI: 10.1073/pnas.0904565106.

26. Lekka M, Gnanachandran K, Kubiak A, Zieliński T, Zemła J. Traction force microscopy – measuring the forces exerted by cells. Micron 2021 -11-01;150:103138. DOI: 10.1016/j.micron.2021.103138.

27. Traction fields, moments, and strain energy that cells exert on their surroundings | american journal of physiology-cell physiology.
